# Glandular cystitis with nephrogenic metaplasia: an unusual finding

**DOI:** 10.1093/omcr/omae184

**Published:** 2025-02-22

**Authors:** Derqaoui Sabrine, Ibrahimi Ahmad, Jahid Ahmed, Zouaidia Fouad, Znati Kaoutar, Nouini Yassine, Bernoussi Zakia

**Affiliations:** Department of Pathology, Ibn Sina Teaching Hospital, Abderrahim Bouabid Avenue, Rabat, Morocco; Faculty of medecine and pharmacy, Mohamed V university Rabat, Abderrahim Bouabid Avenue, Morocco; Faculty of medecine and pharmacy, Mohamed V university Rabat, Abderrahim Bouabid Avenue, Morocco; Department of Urology, Ibn Sina Teaching Hospital, Abderrahim Bouabid Avenue, Rabat, Morocco; Department of Pathology, Ibn Sina Teaching Hospital, Abderrahim Bouabid Avenue, Rabat, Morocco; Faculty of medecine and pharmacy, Mohamed V university Rabat, Abderrahim Bouabid Avenue, Morocco; Department of Pathology, Ibn Sina Teaching Hospital, Abderrahim Bouabid Avenue, Rabat, Morocco; Faculty of medecine and pharmacy, Mohamed V university Rabat, Abderrahim Bouabid Avenue, Morocco; Department of Pathology, Ibn Sina Teaching Hospital, Abderrahim Bouabid Avenue, Rabat, Morocco; Faculty of medecine and pharmacy, Mohamed V university Rabat, Abderrahim Bouabid Avenue, Morocco; Faculty of medecine and pharmacy, Mohamed V university Rabat, Abderrahim Bouabid Avenue, Morocco; Department of Urology, Ibn Sina Teaching Hospital, Abderrahim Bouabid Avenue, Rabat, Morocco; Department of Pathology, Ibn Sina Teaching Hospital, Abderrahim Bouabid Avenue, Rabat, Morocco; Faculty of medecine and pharmacy, Mohamed V university Rabat, Abderrahim Bouabid Avenue, Morocco

**Keywords:** clinical image, uropathology, glandular cyctitits

Nephrogenic metaplasia/adenoma is a relatively uncommon benign urothelial lesion, with a slight male predilection [1]. The pathophysiology remains unclear [2], however it has been reported in the setting of urothelial injury (trauma, calculi, bacillus Calmette-Guérin therapy) [1]. Glandular cystitis is also a reactive inflammatory urothelial lesion characterized by hyperplastic cystically dilated von Brunn nests, lined by glandular cells with or without intestinal metaplasia [3]. Histologically nephrogenic adenoma has three patterns: tubular, cystic and papillary [1]. These structures are lined by cuboidal to columnar cells, with eosinophilic cytoplasm. Hobnail or clear cells may be occasionnaly present. The cytoplasm is pink and occasionally clear [3]. Immunohistochemistry confirms the diagnosis with positivity for cytokeratin 7 (CK7), α-methylacyl-CoA racemase (AMACR) (P504S), PAX2, and epithelial membrane antigen and Pax 8 [1]. These lesions may mimic urothelial carcinoma clinically and endoscopically [1]. Herein we describe a case of 61- year-old man with a previous history bacillus Calmette-Guérin therapy for a high grade urothelial carcinoma with suspicious cystoscopic lesions. Histological examination revealed metaplastic urothelium lined by glandular tall cells arranged in tubular and papillary pattern (Fig. 1A and B), with foci of nephrogenic metaplasia (Fig. 1C). The latter consisted of cuboidal, and hobnail cells with abundant eosinophilic cytoplasm, expressing CK 7 and Pax 8 positive (Fig. 1D and E). The final diagnosis was glandular cystitis with nephrogenic metaplasia. The main differential diagnosis is nephrogenic adenoma-like clear cell carcinoma [1]. In the absence of nuclear atypia, mitosis, necrosis and infiltrative growth pattern, this diagnosis can be ruled out [3]. Other differential diagnoses include, urothelial carcinoma and prostatic adenocarcinoma [3, 4].

**Figure 1:**
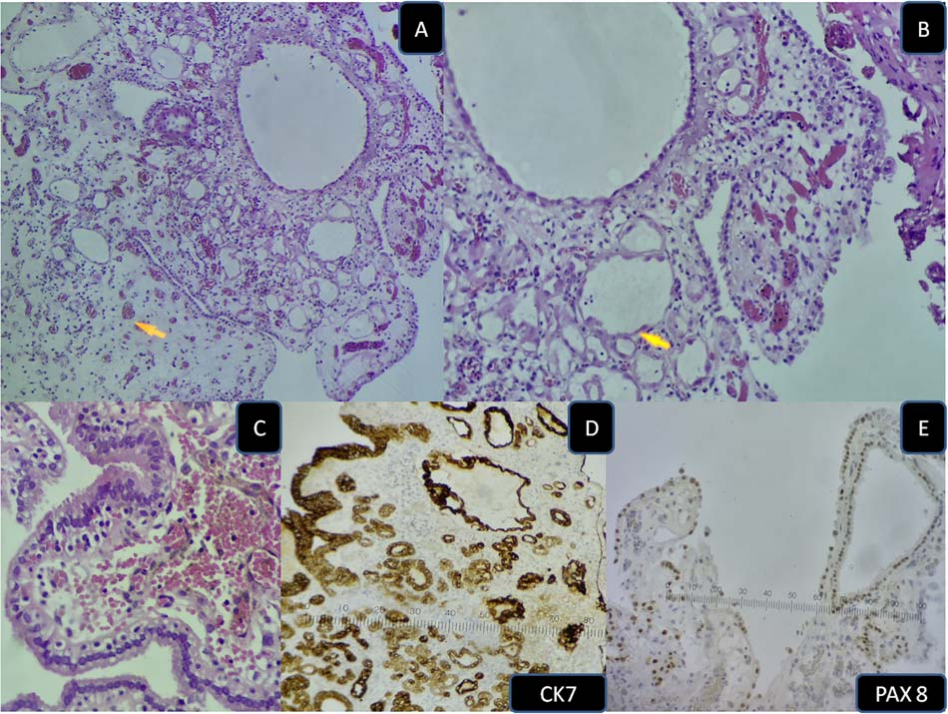
(A) HE low power nephrogenic metaplasia. (B) HE medium power nephrogenic metaplasia. (C) HE high power glandular cystitis. (D) Immunohistochemistry CK 7 positive. (E) Immunohistochemistry Pax8 positive.

In conclusion, nephrogenic adenoma is a benign lesion mimicking malignancy. Careful histological examination with immunohistochemical studies allows for the right diagnosis.

Additionally, 8% of the patients have a previous history of renal transplantation3 or bacillus Calmette-Guérin therapy for urothelial carcinoma of the bladder.

## Data Availability

Not applicable.
